# A new design strategy for redox-active molecular assemblies with crystalline porous structures for lithium-ion batteries†
†Electronic supplementary information (ESI) available: Methods, syntheses, crystallographic data, and supporting figures. CCDC 1887228. For ESI and crystallographic data in CIF or other electronic format see DOI: 10.1039/c9sc04175c


**DOI:** 10.1039/c9sc04175c

**Published:** 2019-11-29

**Authors:** Kensuke Nakashima, Takeshi Shimizu, Yoshinobu Kamakura, Akira Hinokimoto, Yasutaka Kitagawa, Hirofumi Yoshikawa, Daisuke Tanaka

**Affiliations:** a School of Science and Technology , Kwansei Gakuin University , 2-1 Gakuen , Sanda , Hyogo 669-1337 , Japan . Email: yoshikawah@kwansei.ac.jp ; Email: dtanaka@kwansei.ac.jp; b JST , PRESTO , 2-1 Gakuen , Sanda , Hyogo 669-1337 , Japan; c Graduate School of Engineering Science , Osaka University , 1-3 Machikaneyama-cho , Toyonaka , Osaka 560-8531 , Japan

## Abstract

A new design strategy for high-performance organic cathode active materials for lithium-ion batteries is presented, which involves the assembly of redox-active organic molecules with a crystalline porous structure using mixed-stacked charge-transfer (CT) complexes. Hexahydroxytriphenylene was used as a donor molecule and 1,4,5,8,9,12-hexaazatriphenylene-2,3,6,7,10,11-hexacarbonitrile as an acceptor molecule to give a new porous CT complex (**PCT-1**) with a pseudo-hexagonal mixed columnar structure. X-ray diffraction measurements and sorption experiments demonstrated that the intercolumnar spaces in **PCT-1** can incorporate various molecules accompanied by lattice expansion. A lithium metal battery containing **PCT-1** as a cathode active material exhibited a high capacity of 288 mA h g^–1^ at 500 mA g^–1^, and this performance was attributed to a combination of the redox-active units and the porous structure of **PCT-1**.

## Abstract 2

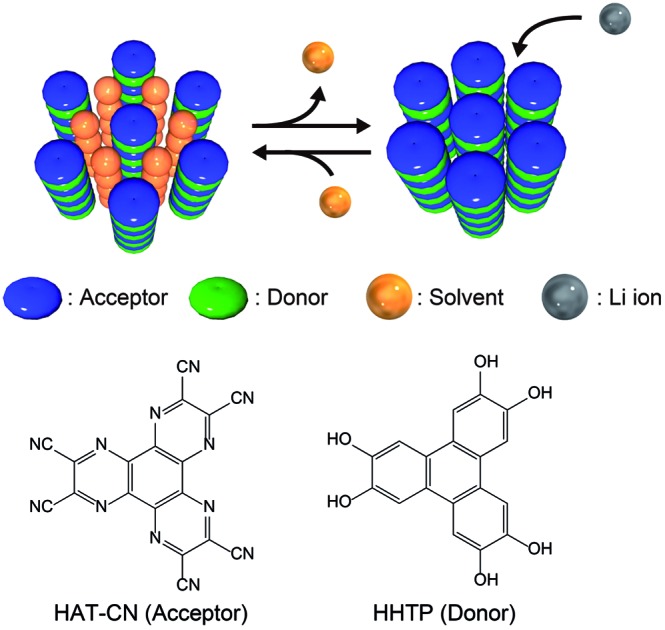
A new design strategy for the high-performance organic cathode-active materials of lithium-ion batteries is presented, which involves the assembly of redox-active organic molecules with a crystalline porous structure.

## Introduction

Lithium-ion batteries (LIBs) exhibit a high energy/power density, high performance, and long life.^1^–^4^ To date, transition metal oxides such as LiMO_2_ (M = Co, Ni, *etc.*), LiM_2_O_4_ (M = Mn, *etc.*), and LiMPO_4_ (M = Fe, Ni, *etc.*) have been utilized as conventional cathode active materials for LIBs.^5^,^6^ However, these inorganic materials contain expensive rare elements and/or exhibit poor rate performance due to the slow intercalation of lithium ions. Therefore, appropriate metal oxide host lattices have been explored to overcome this poor ionic diffusion.^7^ Studies on the application of redox-active organic compounds as cathode active materials have therefore become popular due to the high energy/power densities, low cost, diversity, and structural controllability of these compounds.^8^,^9^ Various organic materials such as organosulfur compounds,^10^ organic carbonyl compounds,^11^–^15^ tetrathiafulvalene derivatives,^16^,^17^ organic nitrogen compounds,^18^–^21^ and organic radical compounds^22^–^24^ have been examined as potential cathode active materials. However, since these materials are molecular crystals assembled *via* weak intermolecular interactions, the formation of robust porous structures is challenging, and instead, densely packed crystal structures in which lithium ions cannot penetrate efficiently are obtained. Recently, crystalline molecular porous materials such as covalent organic frameworks (COFs) and metal–organic frameworks (MOFs) have drawn attention as electrode materials.^25^–^35^ These porous materials can be constructed from redox-active organic ligands and generate a diffusion path for lithium ions by robust coordination and/or covalent bonds, which greatly improves battery performance. However, most MOFs and COFs contain non-redox-active linker units, such as carboxylates and imines, in their open frameworks, which results in lower energy densities,^36^,^37^ whereas some MOFs and COFs have been designed to include redox-active linkers.^27^,^38^,^39^ Despite the high potential of redox-active molecular organic compounds, a universal design strategy for the spontaneous formation of appropriate porous structures for ion diffusion has yet to be discovered to allow the application of such materials as cathode active materials with high rate performance.

**Fig. 1:**
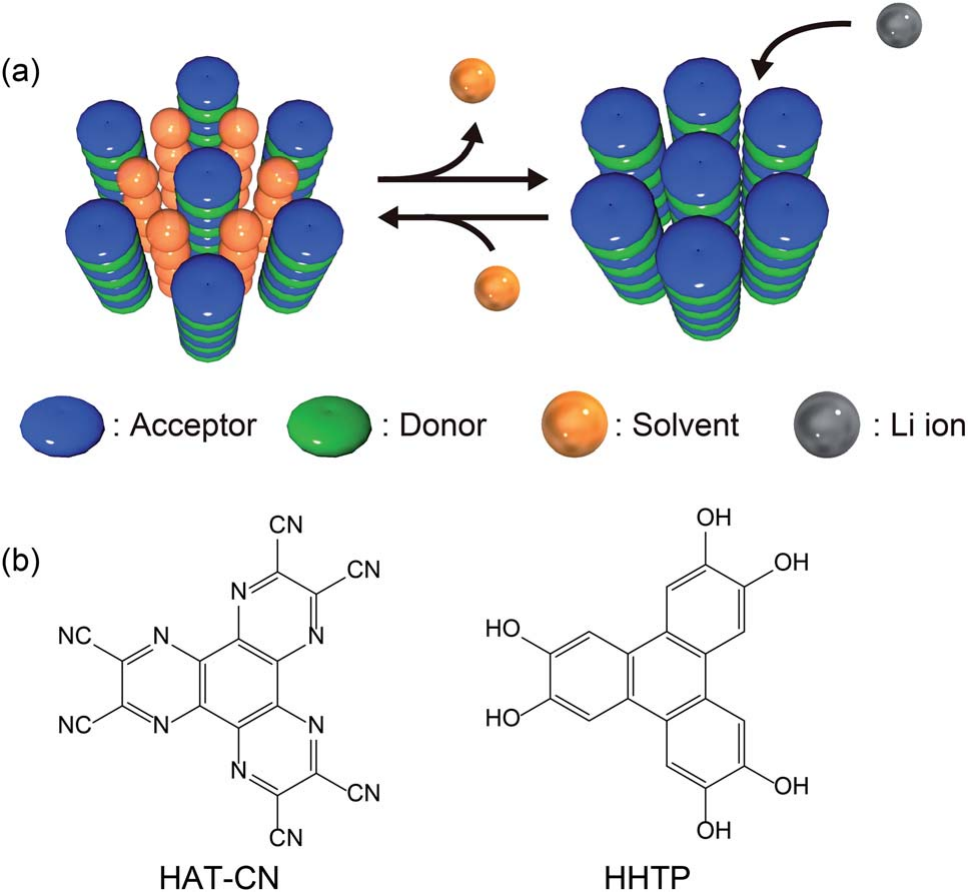
(a) Schematic illustration of the proposed design strategy for redox-active porous structures using CT complexes. (b) Chemical structures of HAT-CN (acceptor) and HHTP (donor).

Herein, we present a new design strategy for the preparation of redox-active molecular assemblies with crystalline porous structures. In recent years, a report into batteries employing organic charge-transfer (CT) complexes as active materials has attracted attention due to the unique redox properties.^40^ Our approach is therefore based on conventional mixed-stacked CT complexes with columnar structures. More specifically, it is known that various electron donor and acceptor aromatic molecule pairs form columnar structures with alternate stacks *via* CT interactions, and that these columns assemble spontaneously to form (pseudo) hexagonal structures (Fig. 1a).^41^–^44^ While redox-active organic molecules densely pack along the columnar direction due to strong CT interactions, we expect that the one-dimensional channels between the columns can be regarded as pores to adsorb various molecules and/or ions.^45^ Since a number of columnar donor–acceptor assemblies have been reported, the demonstration of the porous nature of this structural motif paves the way for a new class of redox-active porous material.

Based on the proposed design strategy, we synthesized a novel CT complex, namely, **PCT-1·DMF**, where “PCT” indicates a porous charge-transfer complex. It has been reported that triphenylene derivatives and hexaazatriphenylene (HAT) derivatives spontaneously form alternate stacking structures.^46^,^47^ **PCT-1·DMF** has a pseudo-hexagonal columnar structure composed of alternately stacked hexahydroxytriphenylene (HHTP) as a donor molecule and 1,4,5,8,9,12-hexaazatriphenylene-2,3,6,7,10,11-hexacarbonitrile (HAT-CN) as an acceptor molecule (Fig. 1b), where the voids between the columns are filled with solvent molecules (*i.e.*, *N,N*-dimethylformamide, DMF). These DMF molecules can be removed by heating *in vacuo*, and the resultant degassed phase, **PCT-1**, maintains its columnar structure. Sorption experiments also demonstrated that the one-dimensional channels present between the columns can include guest molecules accompanied by lattice expansion. HAT derivatives have also been investigated as promising cathode active materials due to their reversible redox reactions.^35^,^48^–^50^ More specifically, the electron-deficient heterocycle core in HAT-CN affords a maximum of six reversible redox reactions,^47^,^49^ and the intercolumnar voids present in **PCT-1** are expected to act as diffusion paths for lithium ions. Furthermore, since HHTP is composed of three conjugated catechol units, a redox reaction involving up to six electrons can be expected. Thus, we prepared a lithium metal battery using **PCT-1** as the cathode active material, and subsequently examined the rate performance of the obtained device.

## Experimental

### Chemicals

All chemicals were purchased from Tokyo Chemical Industry Co., Ltd., Wako Pure Chemical Industries, and Kishida Chemical Co., Ltd. All chemicals and solvents used in the syntheses were of reagent grade and were used without further purification. The procedures employed for samples preparation are detailed in the ESI.†

### Methods

Proton nuclear magnetic resonance (^1^H NMR) spectra were recorded on a JEOL ECX400 (400 MHz) NMR spectrometer. Proton chemical shift values are reported in parts per million (ppm, *δ* scale) downfield from tetramethylsilane, which was used as a reference (*δ* 0). X-ray powder diffraction (XRPD) spectra were recorded on a Rigaku MiniFlex 600 diffractometer at 40 kV and 15 mA using a Cu target tube. Samples were measured without grinding and data were collected at 2*θ* = 3–30° using Cu-K_α_ radiation. Indexing of the XRPD patterns was performed using the Rigaku PDXL XRD analysis software. Scanning electron microscopy (SEM) images were acquired using a JEOL JCM-600Plus NeoScope microscope. Fourier-transform infrared (FT-IR) spectra were recorded on an ATR-FTIR spectrometer (IRAffinity-1S, Shimadzu). Elemental analysis was performed at A-Rabbit-Science Japan Co., Ltd. (an ultra-trace element analysis research center). Water and N_2_ sorption isotherms for **PCT-1** were acquired using a MicrotracBEL BELSORP-max volumetric gas adsorption instrument at 298 and 77 K, respectively. The guest-free phases of **PCT-1** were obtained by treatment under reduced pressure at 120 °C for 12 h. Ultraviolet-visible-near-infrared (UV-Vis-NIR) diffuse reflectance spectroscopy was carried out using a Shimadzu UV-Vis-NIR spectrophotometer (UV-3600) at *λ* = 300–1200 nm with BaSO_4_ as a standard. Further details regarding the computational methods and electrochemical measurements are given in the ESI.†

### Crystal structure determination

Crystallographic data for **PCT-1·DMF** were collected on a CCD diffractometer with Mo-K_α_ radiation. The CrystalClear-SM 1.4 SP1 program (Rigaku) was used for integration of the diffraction profiles. The crystal data were solved by directed methods using the SHELXT program and were refined with SHELXL. Anisotropic thermal parameters were used to refine all non-hydrogen atoms.

## Results and discussion

**Fig. 2:**
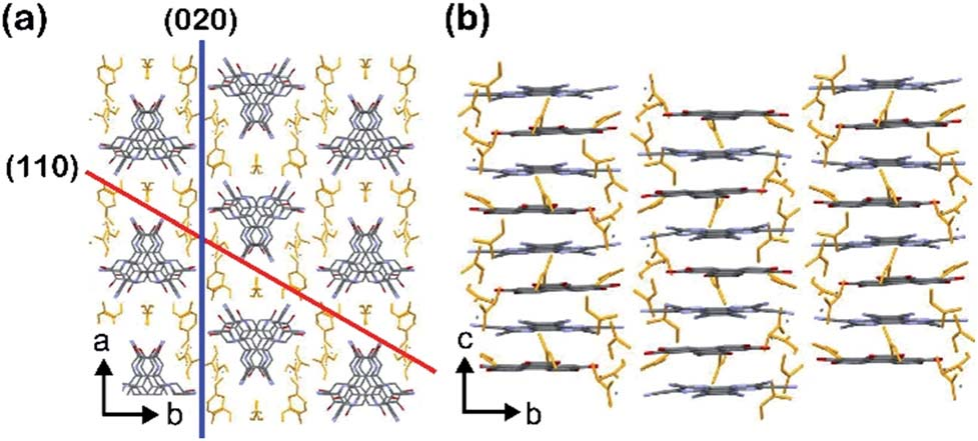
Crystal structure of **PCT-1·DMF**: (a) Top view and (b) side view. The yellow molecules represent DMF. The gray, red, and purple atoms represent C, O, and N, respectively; H atoms are omitted for clarity.

**Fig. 3:**
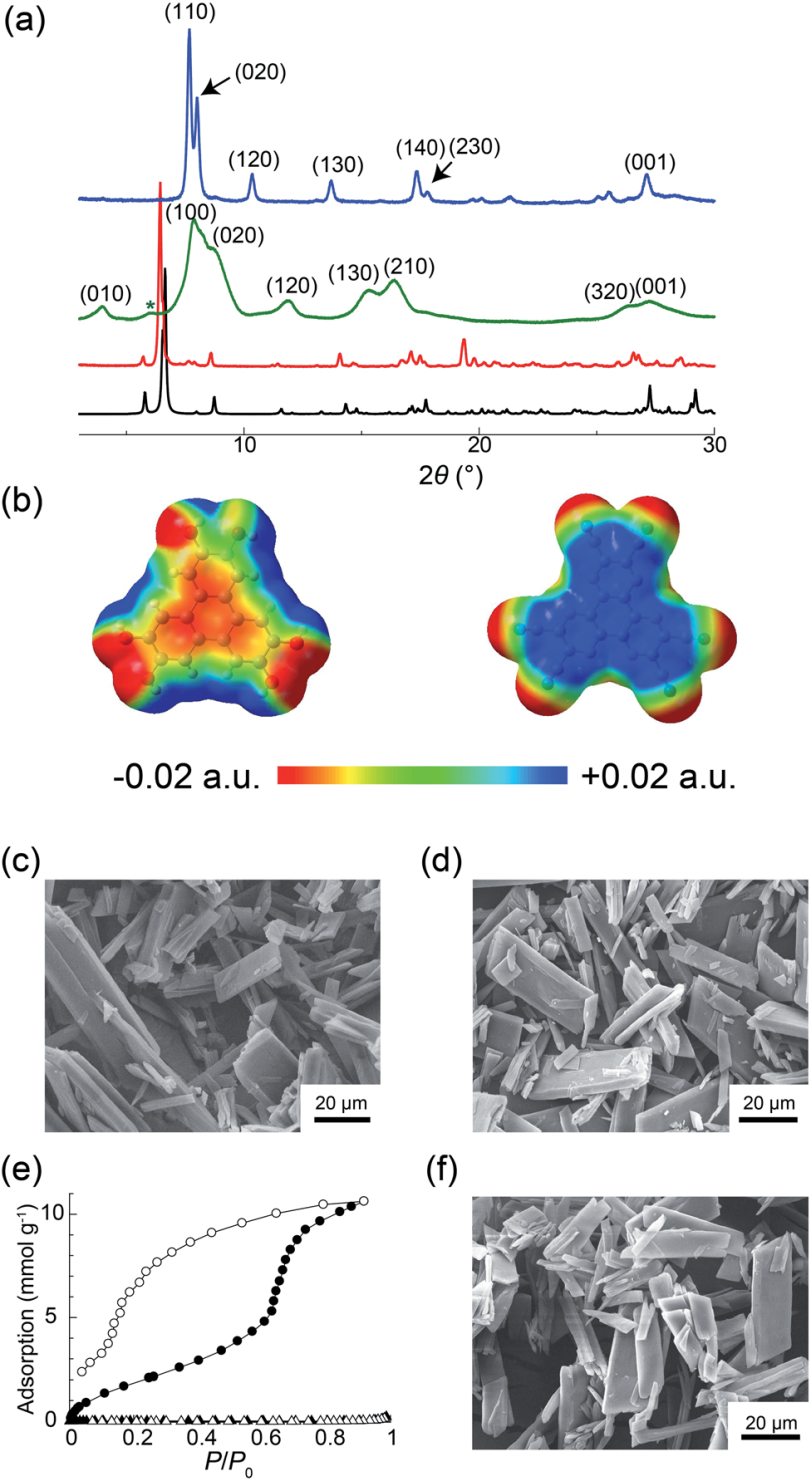
(a) Simulated XRPD pattern of **PCT-1·DMF** (black) and experimental XRPD patterns of **PCT-1·DMF** (red), **PCT-1** (green), and **PCT-1·H_2_O** (blue). The green asterisk indicates the background signal from the Kapton film that shields the sample from ambient air. (b) Electrostatic potentials (ESPs) of HHTP (left) and HAT-CN (right) in the crystal structure of **PCT-1·DMF**. SEM images of (c) **PCT-1·DMF** and (d) **PCT-1**. (e) Adsorption (open symbols) and desorption (solid symbols) isotherms for H_2_O at 298 K (circles) and N_2_ at 77 K (triangles). (f) SEM image of **PCT-1** after water adsorption.

To prepare powder crystalline **PCT-1·DMF**, HHTP and HAT-CN were dissolved in DMF and stirred at 100 °C for 3 h to yield the deep green powder of **PCT-1·DMF**. We also attempted the preparation of a single crystal of **PCT-1·DMF** using various solvent combinations to allow single-crystal X-ray diffraction measurements to be carried out. However, only unsuitable ultrafine needle-like crystals were obtained by the vapor diffusion method at room temperature due to the fast nucleation of **PCT-1·DMF** (Fig. S1 in the ESI†). Thus, to slow the crystallization rate, a boronate ester COF (COF-5) was used as an HHTP source. As the framework of COF-5 is composed of HHTP and boronic acid, and the boronate ester bond easily undergoes hydrolysis, HHTP molecules are expected to be released gradually under ambient conditions, which could in turn control the nucleation rate of **PCT-1·DMF**. Thus, COF-5 and HAT-CN were suspended in DMF and allowed to stand exposed to hexane vapor as a poor solvent. After 1 week, plate-like single crystals of **PCT-1·DMF** with a measurable size of 350 μm were obtained, suggesting that COF-5 was decomposed by moisture in the air and HHTP molecules were released in the DMF solvent. The crystal structure (Fig. 2) revealed that **PCT-1·DMF** exhibits a pseudo-hexagonal columnar structure in which HHTP and HAT-CN molecules stack alternately along the *c* axis. The distance between the π planes of HHTP and HAT-CN in the column was 3.27 Å, suggesting the presence of strong π–π interactions between these two compounds. In addition, the intercolumnar spaces were filled with DMF, which forms hydrogen bonds with the hydroxy groups of HHTP (Fig. S2†). As shown in Fig. 3a, the XRPD pattern of **PCT-1·DMF** agreed with the simulated pattern predicted from the single-crystal structural analysis, demonstrating that the bulk-synthesized dark green powder forms the same structure.

Although the donor HHTP and acceptor HAT-CN compounds are white and yellow powders, respectively, **PCT-1·DMF** was obtained as a deep green powder, suggesting CT-band formation. Indeed, UV-Vis-NIR diffuse reflectance spectroscopy measurements of **PCT-1·DMF** showed a wide absorption band at 700–800 nm (Fig. S3†), which represents the CT band between HHTP and HAT-CN. The electronic state of HAT-CN in **PCT-1·DMF** was then evaluated by FT-IR spectroscopy. It has been reported that the stretching vibration peaks of the nitrile group (*ν*_CN_) of HAT-CN with neutral and anionic radicals appear at 2241 and 2210 cm^–1^, respectively.^51^ In the FT-IR spectrum of **PCT-1**, *ν*_CN_ was observed only at 2241 cm^–1^ (Fig. S4†), indicating that the HAT-CN molecules in **PCT-1·DMF** are neutral. The molecular orbital of an HHTP and HAT-CN dimer cut from the crystal structure of **PCT-1·DMF** was obtained by density functional theory (DFT) calculations using the B3LYP/6-31++G** level of theory under gas-phase conditions including dispersion forces with Grimme's D2 parameter. The highest occupied molecular orbital (HOMO) and lowest unoccupied molecular orbital (LUMO) shown in Fig. S5† confirm that the majority of the HOMO is localized on the HHTP molecule and the majority of the LUMO is localized on the HAT-CN molecule. This result indicates that **PCT-1·DMF** is a neutral CT complex, which is consistent with the FT-IR spectroscopy results. In addition, the electrostatic potentials (ESPs) of the HHTP and HAT-CN molecules were calculated along with the energy of the aromatic interactions (including dispersion forces) to evaluate the strong π–π interactions between HHTP and HAT-CN. The ESPs shown in Fig. 3b demonstrate that the aromatic rings of HHTP and HAT-CN possess negatively and positively charged cores, respectively, suggesting that the mixed columnar structures are stabilized by electrostatic interactions between the oppositely charged aromatic cores. In addition, the energy of the π–π interactions between HHTP and HAT-CN was calculated using the cut dimeric structure, giving an estimated stack energy of –39.0 kcal mol^–1^, which indicates a greater stability than that provided by π–π interactions between HAT-CN (*i.e.*, –13.8 kcal mol^–1^), as estimated from the reported crystal structure of HAT-CN (Table S1†).^52^ Furthermore, in the crystal structure of **PCT-1·DMF**, six DMF molecules form hydrogen bonds to the hydroxy groups of HHTP. The calculated π stack energy using the HHTP and HAT-CN dimer with six molecules of DMF was –66.9 kcal mol^–1^ (Table S2†). The strong π–π interactions can therefore be explained by the fact that hydrogen bonds increase the negative charge of the HHTP aromatic ring and therefore the electrostatic interactions between HAT-CN and HHTP become stronger.

The guest-free phase of **PCT-1** was then prepared by heating **PCT-1·DMF** at 120 °C overnight under reduced pressure. The ^1^H NMR spectrum of **PCT-1** in methanol-*d*_4_ revealed that only one DMF molecule per HHTP molecule remained in the structure, indicating that the other five DMF molecules were removed upon heating (Fig. S6†). CHN elemental analysis also confirmed the existence of one DMF molecule in **PCT-1**. The XRPD pattern of **PCT-1** acquired in a dry Ar atmosphere (Fig. 3a) shows broader peaks than that of **PCT-1·DMF**, in which fewer peaks are observed. The majority of peaks corresponding to **PCT-1** were indexed as (*hk*0), and all peaks were observed to be shifted to larger 2*θ* values. The d-spacing of the peak at 2*θ* = 27.4°, assigned to the (001) diffraction, is 3.26 Å, which corresponds to the distance between the π planes of HHTP and HAT-CN, and indicates that the intermolecular distances in the columns remain constant following DMF removal. Assuming an orthorhombic crystal system, the assigned cell parameters are *a* = 11.2 Å, *b* = 20.6 Å, and *c* = 3.26 Å. When pseudo-hexagonal packing of the columns on the *ab* plane is assumed, a column diameter of approximately 10–11 Å is obtained, which is consistent with the sizes of HAT-CN and HHTP. These results strongly suggest that a structural transformation occurred after DMF removal and that the intercolumnar distance was reduced to maintain the pseudo-hexagonal columnar packing due to the strong π–π interactions between HHTP and HAT-CN. The SEM images shown in Fig. 3c and d indicate that the crystal morphology of **PCT-1** was maintained after DMF removal. Interestingly, the XRPD pattern became sharper and shifted after exposing **PCT-1** to ambient air or water vapor (Fig. 3a). Assuming that the crystal system is orthorhombic, the unit cell parameters are *a* = 13.5 Å, *b* = 22.1 Å, and *c* = 3.29 Å, indicating that the lattice expanded along the direction of the *ab* plane. This result strongly suggests that **PCT-1** rapidly adsorbs water molecules in the hydrophilic intercolumnar spaces to form **PCT-1·H_2_O**. In addition, following exposure of **PCT-1·H_2_O** powder to DMF vapor, the original XRPD pattern of **PCT-1·DMF** was recovered, indicating that the adsorption–desorption process of the guest is reversible and that **PCT-1** can be regarded as a porous material (Fig. S7 and S8†).

To explore the porous properties of **PCT-1** in detail, the water adsorption isotherm was acquired at 298 K. As shown in Fig. 3e, water was adsorbed with a large hysteresis and a step was present at *P*/*P*_0_ = 0.7, which is typically observed in the sorption isotherms of flexible MOFs in which structural transformations occur.^53^–^55^ At *P*/*P*_0_ = 0.92, the adsorption was 238 cm^3^ g^–1^ (at STP), which corresponds to the adsorption of 15 water molecules per HHTP/HAT-CN dimer. This stepwise sorption behavior suggests that the structural transformation or lattice expansion occurred at *P*/*P*_0_ = 0.7. N_2_ adsorption measurements of **PCT-1** at 77 K were also performed, and the obtained N_2_ isotherm showed that **PCT-1** does not adsorb nitrogen at this temperature. This result suggests that **PCT-1** adsorbs only polar molecules (*e.g.*, H_2_O and DMF) in the intercolumnar space or that the structural transformation requires a higher temperature due to the high activation energy necessary for the diffusion process. The XRPD pattern after the water-sorption experiments (Fig. S9†) is similar to that prior to water adsorption. In addition, SEM imaging confirmed that the morphology was maintained after water adsorption (Fig. 3f), suggesting that recrystallization did not occur during the adsorption measurements and water adsorption did not destroy the **PCT-1** columnar structure. These results demonstrate that the pseudo-hexagonal columnar packing is retained during water adsorption.

**Fig. 4:**
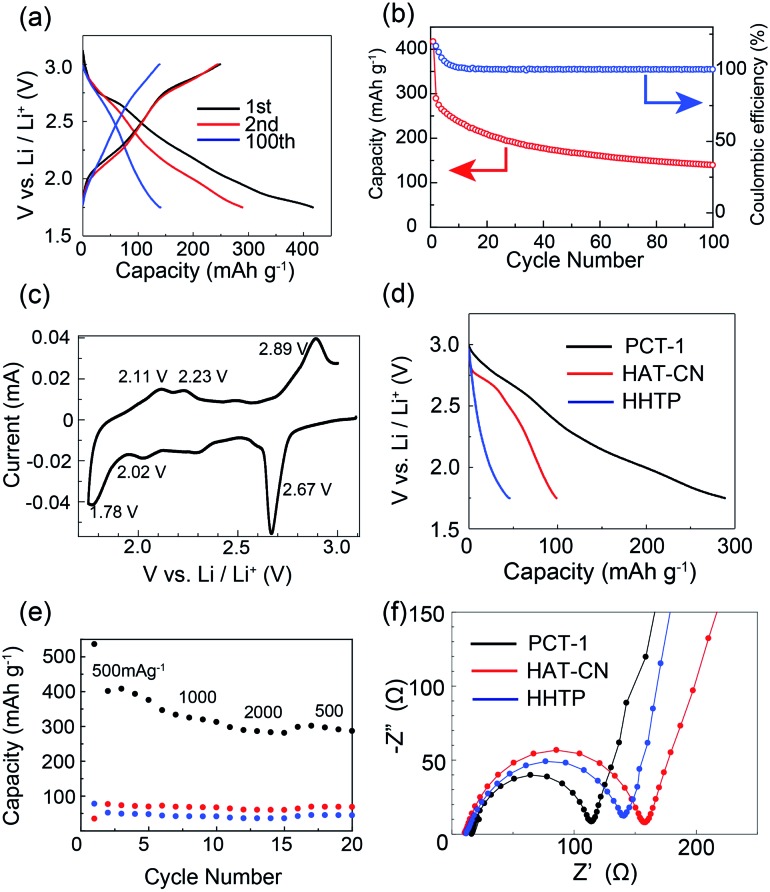
(a) Galvanostatic charge–discharge profiles of **PCT-1** at 500 mA g^–1^. (b) Capacity and coulombic efficiency of the battery. (c) CV profile of **PCT-1** for the first cycle. (d) Galvanostatic charge–discharge profiles of **PCT-1**, HAT-CN, and HHTP at 500 mA g^–1^ for the second cycle. (e) Rate performance of **PCT-1** (black), HAT-CN (red), and HHTP (blue) at 500–2000 mA g^–1^. (f) EIS profiles of **PCT-1**, HAT-CN, and HHTP.

To evaluate the electrochemical performance of **PCT-1**, coin-type lithium metal battery cells were assembled using cathodes with **PCT-1** and lithium foil as anodes. The pellet cathode samples were composed of 30 wt% **PCT-1**, 60 wt% carbon black (to improve electrical conductivity), and 10 wt% PVDF.^22^ Before conducting electrochemical experiments, the solubilities of **PCT-1**, HAT-CN, and HHTP in the electrolyte were checked. While slight elution of **PCT-1** into the electrolyte was observed, HHTP and the HAT-CT monomer were well dissolved under the same conditions, demonstrating that elution was suppressed by CT complex formation. The voltage range used to evaluate the battery performance of the coin-type cells was determined from the cyclic voltammetry (CV) peak position and the reversibility. CV measurements in three voltage ranges (1.0–4.0 V, 1.5–3.5 V, and 1.75–3.0 V) demonstrated that peak reversibility at 2.67 V was observed only when the voltage range was 1.75–3.0 V (Fig. S10†). Therefore, galvanostatic charge–discharge measurements were carried out at voltages of 1.75–3.0 V with a current density of 500 mA g^–1^. Despite using high current density conditions, the discharge capacities in the first and second cycles were 416 and 288 mA h g^–1^, respectively (Fig. 4a), and the numbers of electrons were 11.0 and 7.6 per HHTP/HAT-CN dimer, respectively. The energy densities in the first and second cycles were 151.0 and 73.7 W h kg^–1^, respectively, and the power densities in the first and second cycles were 331.7 and 232.9 W kg^–1^, respectively. As two kinds of multi-redox-active molecules are present in **PCT-1**, *i.e.*, HHTP and HAT-CN, multiple electron reactions can occur. We assume that each HAT-CN and HHTP molecule undergoes an ideal 6e^–^ redox reaction and that the total maximum theoretical capacity of **PCT-1** is 454 mA h g^–1^, which is of the same order as the first discharge capacity. In addition, the high capacity of the lithium metal battery cell under these high current density conditions suggests that lithium ions can pass through the intercolumnar space in **PCT-1**, and therefore, HHTP and HAT-CN molecules inside the crystal lattices react efficiently. Although the capacity of the battery after 100 cycles was 48.1% of that after 2 cycles, the coulombic efficiency was maintained at ∼100% over 100 cycles, and all electric energy obtained in the charge process could be utilized in the discharge process (Fig. 4b). The low cycling performance is therefore attributed to the gradual elution of **PCT-1** into the electrolyte, despite **PCT-1** being stabilized by CT complex formation, as mentioned above. In contrast, the abrupt decrease in capacity in the first cycle, which is sometimes observed in batteries that use molecular cathode active materials, can be ascribed to several factors.^22^,^56^ Possible reasons for this phenomenon include the dissolution of **PCT-1** in the electrolyte, the formation of a solid electrolyte interface, and supercapacitance effects on the electrode surface. In addition, HHTP possesses six hydroxy groups, which can be deprotonated and lithiated under the conditions in the cell. The deprotonation process is irreversible; hence, it may also be responsible for the dramatic capacity decrease observed in the second charge/discharge curve. The properties of a battery using **PCT-1·DMF**, where DMF molecules are present in the intercolumnar spaces, were also examined. The galvanostatic charge–discharge profiles revealed that the discharge capacity in the second cycle was 103 mA h g^–1^ and the number of electrons was 4.4 per HHTP/HAT-CN dimer, which is lower than the performances of degassed **PCT-1** (Fig. S11†). These results suggest that the stable hydrogen bonds between the DMF molecules and hydroxy groups prevent Li^+^ ion diffusion. In contrast, when the amount of **PCT-1** in the cathode was increased or the thickness of the cathode was increased, the cell capacities decreased, likely due to the low electrical conductivity of the alternately stacked CT complex, **PCT-1** (Fig. S12 and S13†). The capacity of a battery without the active material (only the carbon additive) was quite low (<14 mA h g^–1^), indicating that the contribution the carbon additive is limited or negligible (Fig. S14†).

To investigate the electrochemical behavior of **PCT-1** in the cell, CV measurements were conducted on the fabricated cell. As shown in Fig. 4c, the cathodic peaks at 1.78, 2.02, and 2.67 V (*vs.* Li^+^/Li) represent the reduction of **PCT-1** and the insertion of lithium ions, while the anodic peaks at 2.11, 2.23, and 2.89 V (*vs.* Li^+^/Li) are attributable to the oxidation and extraction of lithium ions, demonstrating that **PCT-1** exhibits a reversible multistep redox ability. The three redox potentials, *E*_1/2_, at 1.95, 2.13, and 2.76 V (*vs.* Li^+^/Li) are close to the redox potentials of HHTP and HAT-CN in the coin-type cell, as shown in Fig. S15,† which suggests that the redox reaction of HHTP and HAT-CN influences battery performance. The redox reaction of 5,6,11,12,17,18-hexaazatrinaphthylene (HATNA), a HAT derivative, was reported to be a 6e^–^ reaction that can be divided in two three-electron processes, *i.e.*, 2.6–2.15 V and 1.68–1.38 V.^49^ Since HAT-CN has a lower LUMO level than HATNA, the redox reaction of HAT-CN occurs in the higher voltage region, which is consistent with the CV profile of **PCT-1**. The redox peaks remained constant after 10 CV cycles, although the peak intensity decreased, which is consistent with the low cycle performance of the battery capacity (Fig. S10†). The capacitive contributions in the CV profiles were separated from the total current according to the reported method (Fig. S16†).^57^ The voltage profile for the capacitive current demonstrates that the anodic peaks at 2.11, 2.23, and 2.89 V (*vs.* Li^+^/Li) and the cathodic peaks at 1.78, 2.02, and 2.67 V (*vs.* Li^+^/Li) do not originate from the capacitive effect. In contrast, the large capacity and the sloping potential of the battery are partly due to the relatively large capacitive contribution, which was attributed to the porous structure of **PCT-1**. Furthermore, the XRPD patterns of the cathode before and after the charge and discharge processes demonstrate that the hexagonal packing structure was retained, although the peaks became broader after charge–discharge (Fig. S17†).

To compare the CT complex with the HHTP and HAT-CN monomers, we conducted a comparative battery performance experiment using cathodes containing only HHTP or HAT-CN molecules. At 500 mA g^–1^, the discharge capacities in the second cycle for **PCT-1**, HHTP, and HAT-CN were 288, 45, and 98 mA h g^–1^, respectively, as shown in Fig. 4d and S18.† The capacity of **PCT-1** was significantly higher than those of the monomers, suggesting that the stable one-dimensional mixed columnar structure enhances the stability and the efficient diffusion of lithium ions. The CV profile of the HHTP monomer showed broad redox peaks and the battery performance was rather low, although the high capacity and CV peak at 2.67 V (*vs.* Li^+^/Li) of **PCT-1** suggested the existence of an HHTP redox reaction in the CT complex, which could account for the large capacity of **PCT-1**. The high redox activity of HHTP in **PCT-1** is most likely due to the porous structure and the efficient diffusion of Li^+^ ions in the crystal lattice.

The rate performance of **PCT-1**, HHTP, and HAT-CN shown in Fig. 4e reveals that the high capacity of **PCT-1** is retained even under rapid charge and discharge conditions. This result suggests that the intercolumnar channels in **PCT-1** enhance the efficient diffusion of lithium ions, thereby improving the rate performance. The galvanostatic intermittent titration technique (GITT) was used to evaluate the diffusion rate of lithium ions in **PCT-1** (Fig. S19†). The diffusion coefficient (*D*_Li^+^_) of lithium ions calculated by the GITT was 6.73 × 10^–11^ cm^2^ s^–1^, which is comparable to those of conventional metal oxides and/or highly porous materials such as exfoliated COFs.^32^,^58^ The high *D*_Li^+^_ of **PCT-1** was therefore attributed to the channels between the columnar structures, which act as diffusion paths for lithium ions. To investigate the CT resistance in the active material, electrochemical impedance spectroscopy (EIS) measurements were carried out. As shown in Fig. 4f and S20,† the semicircles in the Nyquist plots and the equivalent circuit fitting results demonstrate that the CT resistance of **PCT-1** (96 Ω) is lower than those of HAT-CN (146 Ω) and HHTP (131 Ω), suggesting that the CT resistance was also improved by the formation of columnar structures.

## Conclusions

In conclusion, we successfully synthesized a new neutral charge-transfer (CT) complex, **PCT-1**, composed of hexahydroxytriphenylene (HHTP) as a donor molecule and 1,4,5,8,9,12-hexaazatriphenylene-2,3,6,7,10,11-hexacarbonitrile (HAT-CN) as an acceptor molecule. X-ray powder diffraction and sorption experiments revealed that the intercolumnar voids can incorporate various molecules *via* lattice expansion. A lithium metal battery with **PCT-1** as a cathode active material exhibited a high capacity and fast charge–discharge properties, which were attributed to the combination of redox-active units and the porous structure of **PCT-1**. In addition, CT complex formation stabilized the organic molecules in the electrolyte and is a new method for suppressing the elution of organic compounds into the electrolyte, which is one of the problems associated with organic active materials. As various donor–acceptor pairs can form mixed columnar structures with hexagonal packing, it is apparent that our strategy of using CT complexes has the potential to be applied to porous electrode active materials. However, as **PCT-1** requires a relatively high amount of carbon additive, new CT complexes with higher conductivities need to be developed for future applications. We expect that this concept can be extended to other compounds; hence, our potentially universal design strategy for porous redox-active assembled structures opens up a new class of porous material, namely, CT complexes with high electrical conductivities and high stabilities, for use in appropriate electrolytes. A study into the cathode active material properties of various CT complexes with mixed columnar structures is currently underway in our laboratory and the results will be presented in due course.

## Conflicts of interest

There are no conflicts to declare.

## Supplementary Material

Supplementary information

Crystal structure data
