# Supplementary material for: A new design strategy for redox-active molecular assemblies with crystalline porous structures for lithium-ion batteries
Source: Chem Sci. 2019 Nov 29;11(1):37–43. doi: 10.1039/c9sc04175c (PMC7067258; doi:10.1039/c9sc04175c)
Supplement: Supplementary file 1 [file SC-011-C9SC04175C-s001.pdf]

Electronic Supplementary Material (ESI) for Chemical Science.
This journal is © The Royal Society of Chemistry 2019

Electronic Supplementary Information

A new design strategy for redox-active molecular assemblies with crystalline porous structures for lithium-ion batteries

Kensuke Nakashima,† Takeshi Shimizu,† Yoshinobu Kamakura, Akira Hinokimoto, Yasutaka Kitagawa, Hirofumi Yoshikawa, and Daisuke Tanaka**

†These authors contributed equally to this work.

Abstract: A new design strategy for high-performance organic cathode active materials for lithium-ion batteries is presented, which involves the assembly of redox-active organic molecules with a crystalline porous structure using mixed-stacked charge-transfer (CT) complexes. Hexahydroxytriphenylene was used as a donor molecule and 1,4,5,8,9,12-hexaazatriphenylene-2,3,6,7,10,11-hexacarbonitrile as an acceptor molecule to give a new porous CT complex (**PCT-1**) with a pseudo-hexagonal mixed columnar structure. X-ray diffraction measurements and sorption experiments demonstrated that the intercolumnar spaces in **PCT-1** can incorporate various molecules accompanied by lattice expansion. A lithium metal battery containing **PCT-1** as a cathode active material exhibited a high capacity of 288 mAh g^{-1} at 500 mA g^{-1}, and this performance was attributed to a combination of the redox-active units and the porous structure of **PCT-1**.

Table of Contents

S1. Methods

S2. Syntheses of materials

S3. Crystallographic data

S4. Supporting figures

S1. Methods

Computational method. The DFT calculations were performed using the B3LYP/6-31++G** level of theory under gas-phase conditions including dispersion forces with Grimme's D2 parameter.[1] The total energy and ESP were calculated using the crystal structure of **PCT-1·DMF** without molecular structure optimization. All calculations were performed using Gaussian 16 packages.[2] The π–π interactions between HHTP and HAT-CN (E_{int}) can be calculated according to Eq. 1:

$$E_{int} = E_{dimer} - (E_{HHTP} + E_{HAT}) \quad \text{Eq. 1}$$

where E_{dimer}, E_{HAT}, and E_{HHTP} represent the calculated total energies of the HHTP/HAT-CN dimer, HHTP, and HAT-CN, respectively.

Table S1. Total energies of HHTP, HAT-CN, and the HHTP/HAT-CN dimer and the energy difference between the dimer and monomers

E_{HHTP}	E_{HAT}	E_{dimer}	$E_{int} = E_{dimer} - (E_{HHTP} + E_{HAT})$
−718092.3 kcal mol^{-1}	−842680.2 kcal mol^{-1}	−1560811.5 kcal mol^{-1}	−39.0 kcal mol^{-1}

The π–π interactions between HHTP and HAT-CN containing six DMF molecules ($E_{int·DMF}$) can be calculated according to Eq. 2:

$$E_{int·DMF} = E_{dimer+6DMF} - (E_{HHTP+6DMF} + E_{HAT}) \quad \text{Eq. 2}$$

where $E_{dimer+6DMF}$ and $E_{HHTP+6DMF}$ represent the calculated total energies of the HHTP/HAT-CN dimer with six DMF molecules and HHTP with six DMF molecules, respectively.

Table S2. Total energies of HHTP with six DMF molecules, HAT-CN, and the HHTP/HAT-CN dimer with six DMF molecules and the energy difference between the dimer and monomers

$E_{HHTP+6DMF}$	E_{HAT}	$E_{dimer+6DMF}$	$E_{int·DMF} = E_{dimer} - (E_{HHTP} + E_{HAT})$
−1653502.9 kcal mol^{-1}	−842680.2 kcal mol^{-1}	−2496250.0 kcal mol^{-1}	−66.9 kcal mol^{-1}

Electrode fabrication. The cathode was fabricated as follows. **PCT-1** and carbon black (Toka Black 5500, Tokai Carbon) were mixed and stirred using an agate mortar and a pestle for 30 min. Subsequently, a polyvinylidene fluoride (PVDF, #1100, Kishida Chemical) binder was added and the mixture was stirred for 1 h. The weight ratio of **PCT-1**, carbon black, and PVDF was 30:60:10. The weight of active material per cathode was typically 0.2 mg. The weight of the electrode was measured using a PerkinElmer AD6 autobalance with 0.1 µg resolution. Anhydrous *N*-methylpyrrolidone (NMP) was then added and the resulting mixture stirred for 2 h to give a slurry. This slurry was cast onto 20 µm aluminum foil using a 50 µm doctor blade and dried overnight under vacuum. Then, the foil was cut into a disc with a diameter φ of 15.95 mm.

Carbon-coated separator fabrication. A carbon-coated separator was used to suppress the diffusion of active materials into the anode. Super-P (Alfa Aesar) and acetylene black (Denka Black, Li-100) were mixed and stirred using an agate mortar and pestle for 30 min. Subsequently, the PVDF binder was added and the mixture was stirred for 1 h. The weight ratio of Super-P, acetylene black, and PVDF was 80:10:10. Anhydrous NMP was then added and the resulting mixture stirred for 30 min to give a slurry. This slurry was cast onto a 20 µm polypropylene separator using a 100 µm doctor blade. Then, the separator was cut into a disc with a diameter φ of 17.00 mm.

Battery assembly. Coin cells were assembled in an Ar-filled glovebox using a cathode, lithium foil (thickness: 0.2 mm, diameter φ: 15.5 mm) as the anode, a carbon-coated separator, and 1 M $LiPF_6$ in ethylene carbonate (EC)/diethyl carbonate (DEC) (1:1, v/v) as the electrolyte. Coin cells were prepared similarly for HHTP and HAT-CN.

Cyclic voltammetry (CV). CV measurements (voltage: 1.75–3.0 V, scan rate: 0.2 mV s^{-1}) were carried out using CR2032 coin-type cells on a SP-150 potentiostat (Biologic Science Instruments) at room temperature.

Electrochemical impedance spectroscopy (EIS). EIS measurements (alternating voltage: 5.0 mV, frequency: 1 MHz to 10 mHz) were carried out using CR2032 coin-type cells with initial voltage of 3.102 V on SP-150 potentiostat (Biologic Science Instruments) at room temperature. Coin cells before discharge–charge measurements were used for EIS.

Discharge–charge measurements. Galvanostatic discharge–charge tests (voltage: 1.75–3.0 V, current density: 500, 1000, or 2000 mA g^{-1}) were performed at room temperature on a Hokuto Denko HJ1020mSD8 charging/discharging device. The specific capacity was calculated based on the mass of each active material in the cathode. Because the discharge curves were acquired first, the coulombic efficiency in cycle *n* (CE(*n*)) was calculated using Eq. 3:

$$\mathrm{CE}(n) = \mathrm{Capacity}_{discharge}(n)/\mathrm{Capacity}_{charge}(n\text{-}1) \times 100\ (\%) \quad \text{Eq. 3}$$

where $\text{Capacity}_{\text{discharge}}(n)$ is the discharge capacity in cycle *n* and $\text{Capacity}_{\text{charge}}(n-1)$ is the charge capacity in cycle *n-1*.

Galvanostatic intermittent titration technique (GITT) analysis for Li^+ diffusion coefficient (D_{Li+}) calculations. GITT measurements were carried out using a Hokuto Denko HJ1020mSD8 charging/discharging device. For the measurements, the electrodes were discharged at a constant current density of 0.1 C, corresponding to 45.42 mA g^{-1}, for 600 s, followed by an open circuit relaxation of τ (4800 s) to allow the cell voltages to reach their steady-state voltages. This procedure was repeated in the voltage range of 1.75–3.0 V. The Li^+ diffusion coefficient D_{Li+} can be calculated using Eq. 4.

$$D_{\mathrm{Li}^+} = \frac{4}{\pi\tau}\left[\frac{mV_M}{MS}\right]^2\left[\frac{\Delta E_{\mathrm{s}}}{\Delta E_{\mathrm{t}}}\right]^2\left(\tau \ll \frac{L^2}{D_{\mathrm{Li}^+}}\right) \text{ Eq. 4}$$

where ΔE_s is the change in the steady-state (equilibrium) voltage at the end of two sequential open-circuit relaxation periods, ΔE_t is the total change in the cell voltage during the current pulse (neglecting the IR drop), *M* [g mol^{-1}] is the molecular weight, *m* [g] is the mass of the active material, V_M [m^3 mol^{-1}] is the molecular volume, and *S* [m^2] is the electrode area. In addition, $(mV_M)/MS$ gives the particle size of the active material, corresponding to the electrode thickness *L*, as a diffusion length, and thus the GITT formula can be written as shown in Eq. 5.[3–6]

$$D_{\mathrm{Li}^+} = \frac{4L^2}{\pi\tau}\left[\frac{\Delta E_{\mathrm{s}}}{\Delta E_{\mathrm{t}}}\right]^2\left(\tau \ll \frac{L^2}{D_{\mathrm{Li}^+}}\right) \text{ Eq. 5}$$

Capacitive contribution. CV measurements were carried out to investigate the variation in the total current density *i* at each sweep rate (0.2, 0.5, 1.0, 2.0, 5.0, and 1.0 mV s^{-1}) using a multi electrochemical measurement system HZ-Pro S12 (Hokuto Denko). The following equation was applied to divide the total current density *i* at a sweep rate of 0.2 mV s^{-1} into capacitive i_c and faradaic i_f components. For analytical purposes, Eq. 6 was rearranged to give Eq. 7.

$$i = i_{\mathrm{c}} + i_{\mathrm{f}} = ai + bi = k_1 v + k_2 v^{1/2} \text{ Eq. 6}$$

$$i/v^{1/2} = k_1 v^{1/2} + k_2 \text{ Eq. 7}$$

where *ai* and k_1v correspond to the current contributions from the capacitive current i_c and *bi* and $k_2v^{1/2}$ to those from the faradaic current i_f.[7] Under the hypothesis that the total current densities, *a*, *b*, k_1, and k_2 are constant at 0.05 V, the parameters k_1, k_2, *a,* and *b* were determined at each voltage by solving the following simultaneous equations:

$$\begin{cases} a + b = 1 \\ ai : bi = k_1 v : k_2 v^{1/2} \end{cases} \quad \text{Eq. 8}$$

$$a = \frac{(k_1/k_2)v^{1/2}}{1+(k_1/k_2)v^{1/2}} \quad \text{Eq. 9}$$

Using the obtained *a* values, the capacitive currents were calculated every 0.05 V. Finally, the total current density *i* and the smoothed capacitive current i_c were plotted.

Calculations of energy density and power density. The energy density *E* [Wh kg^{-1}] can be simply calculated using Eq. 10:

$$E = \int C\, dV \quad \text{Eq. 10}$$

The capacity *C* [mAh g^{-1}] and the voltage *V* [V vs Li^+/Li] were obtained from the charge–discharge measurements, and *C* was calculated based on the active material.
The power density *P* [W kg^{-1}] can be obtained as:

$$P = E/t \quad \text{Eq. 11}$$

where *t* [h] is the discharge time.

***Ex situ* XRPD measurements.** *Ex situ* XRPD measurements were performed on a MiniFlex 600 diffractometer (Rigaku) within a 2θ range of 3°–30° at a scan speed of 2° min^{-1} and 12 scans were accumulated. The voltage and current were set to 40 kV and 15 mA, respectively. The XRPD patterns were obtained as an average of four sequential measurements. The pellet cathode samples were composed of 30 wt% **PCT-1**, 60 wt% carbon black, and 10 wt% PVDF. The electrolyte consisted of 1 M $LiPF_6$ in EC/DEC (1:1, v/v) and sheets of carbon-coated separator were employed. *Ex situ* cathodes were taken from CR2032 coin-type cells stopped at 1.75 and 3.0 V during the discharge–charge tests (current density: 50 mA g^{-1}), and then dried overnight under vacuum.

S2. Syntheses of materials

Synthesis of 2,3,6,7,10,11-hexamethoxytriphenylene (HMTP)

HMTP was synthesized according to a previous report.[8] Dimethoxybenzene (7.8 mL, 61 mmol) was added to dry dichloromethane (300 mL) and nitrogen was bubbled through the solution for 30 min. With the mixture still under nitrogen, anhydrous $FeCl_3$ (26.5 g) was added slowly over 10 min under a heated vacuum and the resulting mixture was allowed to stir at room temperature for 90 min. Subsequently, the reaction mixture was poured into methanol (450 mL) and allowed to settle overnight in a refrigerator. The obtained solid was filtered and washed with large amounts of methanol and ethanol prior to vacuum drying overnight to yield a white powder (5.29 g, 63.7%); ^{1}H NMR (400 MHz, $CDCl_3$) δ 4.13 (s, 18H), 7.83 (s, 6H).

Synthesis of 2,3,6,7,10,11-hexahydroxytriphenylene (HHTP)

HHTP was synthesized according to a previous report.[8] 2,3,6,7,10,11-Hexamethoxytriphenylene (2.50 g, 6.1 mmol) was suspended in a 47% HBr/AcOH solution (1:1, v/v, 150 mL) and the suspension was stirred under nitrogen at room temperature for 30 min prior to heating at reflux for 24 h. After allowing the mixture to cool to room temperature, the obtained precipitate was filtered, washed with excess water, and then dissolved in a hot AcOH/H_2O solution (3:2, v/v, 200 mL) before adding activated carbon. After filtration, the solvents were evaporated to dryness, and the resulting solid was recrystallized from acetone/hexane, filtered, and vacuum dried overnight to yield a white powder (0.897 g, 45.2%); ^{1}H NMR (400 MHz, acetone-d_6) δ 7.60 (s, 6H), 9.27 (s, 6H).

Synthesis of 1,4,5,8,9,12-hexaazatriphenylene-2,3,6,7,10,11-hexacarbonitrile (HAT-CN)

HAT-CN was synthesized according to a previous report.[9] Hexaketocyclohexane octahydrate (11.93 g, 38 mmol) and diaminomaleonitrile (31.58 g, 292 mmol) were dissolved in AcOH (500 mL). This solution was then heated at reflux for 2 h under nitrogen, and the resulting dark brown-black mixture was filtered and washed with hot AcOH (350 mL). The obtained black solid was suspended in 30% HNO_3 (40 mL) and heated to 100 °C for 3 h, after which time the crude product was added to ice water and filtered. Following recrystallization from acetone/hexane, the obtained compound was filtered and vacuum dried overnight to yield a yellow-brown powder (8.58 g, 58.5%).

Synthesis of 1,4-phenylenebis(boronic acid) (PBBA)

PBBA was synthesized according to a previous report.[10] *p*-Dibromobenzene (7.47 g, 32 mmol) was dissolved in dry THF (32 mL). This solution was added to a suspension of magnesium (2.23 g, 92 mmol) in dry THF (16 mL), and the mixture was heated at reflux for 15 h under a nitrogen atmosphere. After cooling the resulting mixture to room temperature, a solution of $B(OMe)_3$ (14.2 mL, 127 mmol) in dry THF (16 mL) was added dropwise at −70 °C and the suspension was stirred under nitrogen at −70 °C for 4.5 h. Subsequently, 2 M HCl aq. (150 mL) was added and the resulting mixture was stirred at room temperature for 1 h prior to extraction with diethyl ether (200 mL × 4) and drying of the organic layer over anhydrous Na_2SO_4. After filtration, the solvents were evaporated to dryness, and the resulting solid was dissolved in hot water. The solution was concentrated in vacuo and the obtained compound was filtered, washed with cold water, and then vacuum dried overnight to yield a white powder (2.23 g, 43.0%); ^{1}H NMR (400 MHz, DMSO-d_6) δ 7.73 (s, 4H), 8.01 (s, 4H).

Synthesis of COF-5

COF-5 was synthesized according to a previous report.[11] HHTP (0.518 g, 1.6 mmol), PBBA (0.418 g, 2.5 mmol), and dry MeOH (0.1 mL) were dissolved in a dioxane/mesitylene solution (4:1, v/v, 16 mL) and nitrogen was bubbled through the solution for 30 min. Subsequently, the solution was heated to 90 °C for 20 h and then cooled to room temperature. The obtained solid was isolated by filtration and washed with toluene (×3). Finally, the solid was soaked overnight and then vacuum dried overnight to yield an off-white powder (0.599 g, 80.0%).

Synthesis of PCT-1·DMF

HHTP (0.897 g, 2.8 mmol) and HAT-CN (1.087 g, 2.8 mmol) were dissolved in DMF, and the resulting solution was stirred at 100 °C for 3 h. After cooling to room temperature, the solid was isolated by filtration to yield a deep green powder (2.549 g, 79.4%).

Preparation of PCT-1

PCT-1·DMF was treated under reduced pressure at 120 °C overnight to yield **PCT-1**. Elemental analysis: calcd for **PCT-1**·(DMF)$(H_2O)_4$ ($C_{39}H_{27}N_{13}O_{11}$) (%): C 54.87, H 3.19, N 21.33; found: C 54.85, H 3.00, N 21.22.

Preparation of a single crystal of PCT-1·DMF

A mixture of HAT-CN dissolved in DMF and COF-5 suspended in DMF was allowed to stand for 1 week under vapor-diffusion conditions with hexane as a poor solvent. A plate-like single crystal was obtained.

S3. Crystallographic data (CCDC: 1887228)

Compound	**PCT-1·DMF**
Formula	$C_{54}H_{54}N_{18}O_{12}$
Formula weight	1147.15
Crystal system	Monoclinic
Space group	$P2_1/c$
a (Å)	16.615(4)
b (Å)	27.205(5)
c (Å)	13.426(3)
β (°)	112.678(4)
V (Å^3)	5600(2)
Z	4
Temperature (K)	200
GOF	0.995
R_1 [$I > 2\sigma(I)$]	0.0619
wR_2 [$I > 2\sigma(I)$]	0.1931

S4. Supporting figures

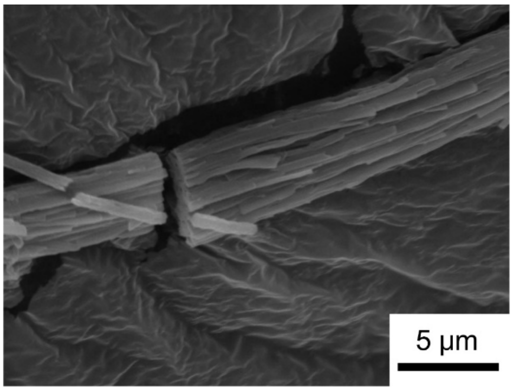

Figure S1. SEM image of polycrystalline **PCT-1·DMF**.

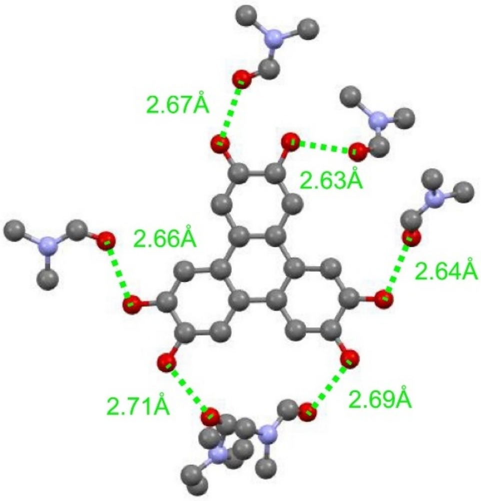

Figure S2. Hydrogen bond distances between HHTP and DMF. The gray, red, and purple atoms represent C, O, and N, respectively; H atoms are omitted for clarity.

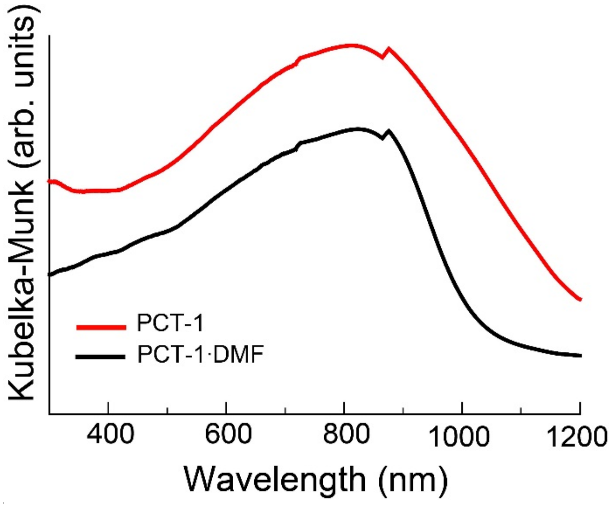

Figure S3. UV-Vis-NIR diffuse reflectance spectra of **PCT-1·DMF** and **PCT-1**.

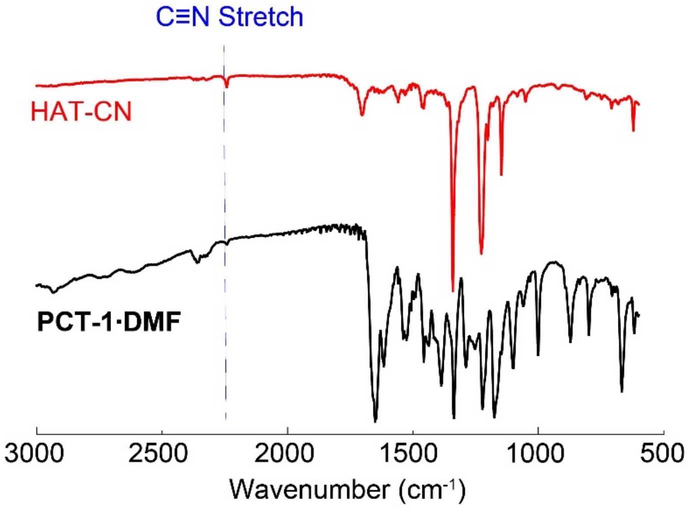

Figure S4. FT-IR spectra of **PCT-1·DMF** and HAT-CN.

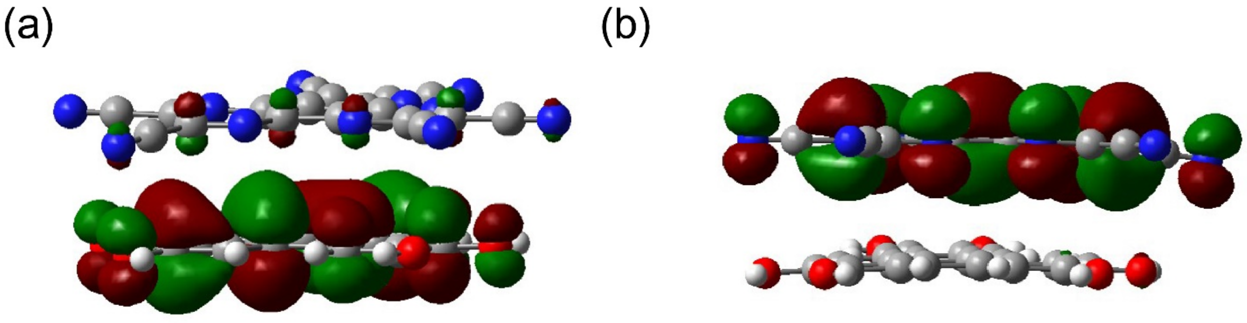

Figure S5. (a) HOMO and (b) LUMO diagrams of the HHTP/HAT-CN dimer cut from the crystal structure of **PCT-1·DMF**.

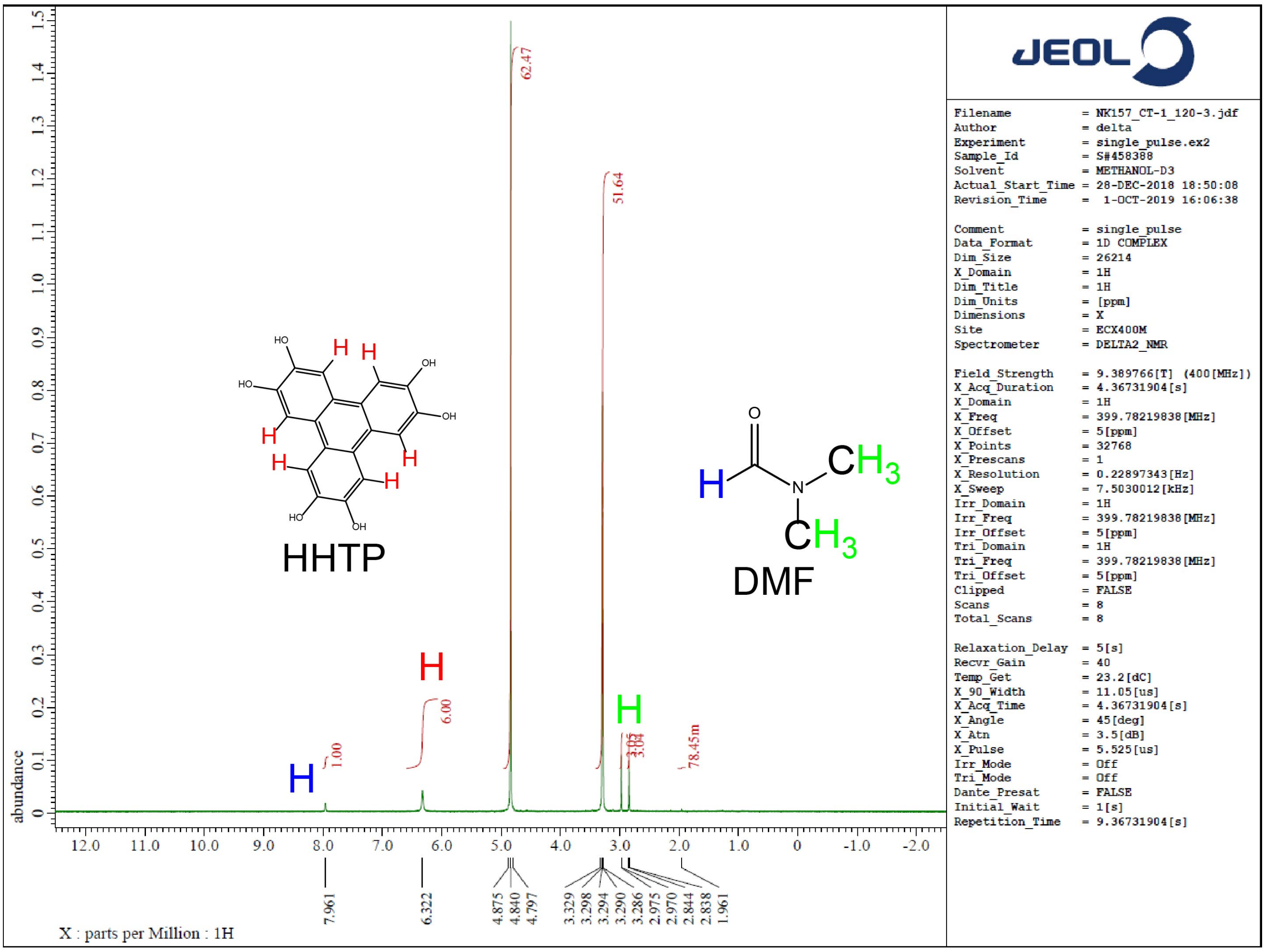

Figure S6. ^{1}H NMR (400 MHz, MeOH-d_4) spectrum of **PCT-1**.

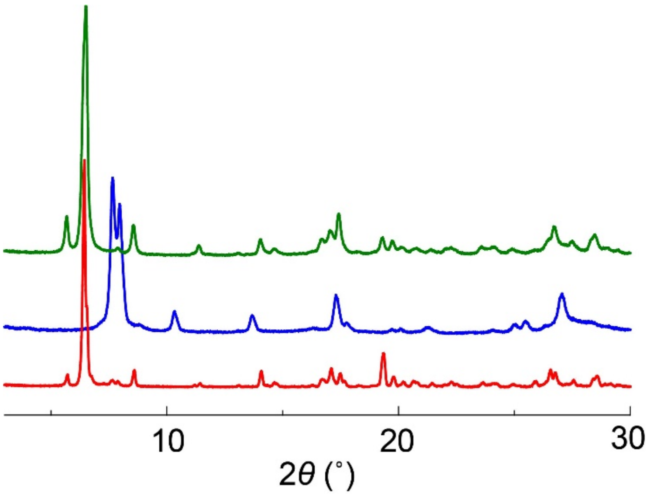

Figure S7. XRPD patterns of **PCT-1·DMF** (red), **PCT-1·H_2O** (blue), and **PCT-1** after exposure to DMF (green).

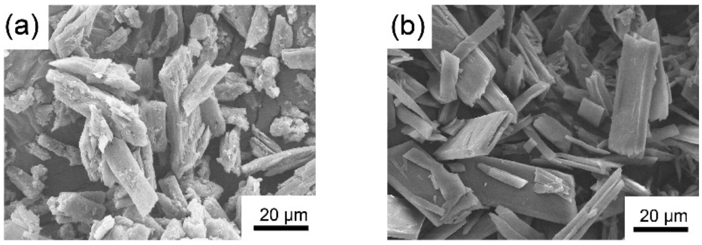

Figure S8. SEM images of **PCT-1** after (a) exposure to DMF and (b) N_2 adsorption.

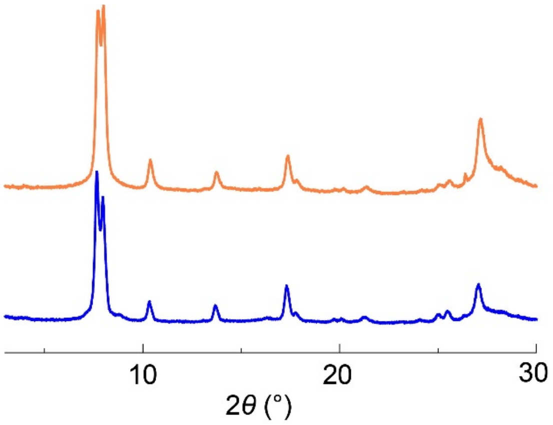

Figure S9. XRPD patterns of **PCT-1·H_2O** before (blue) and after (orange) the H_2O adsorption isotherm experiments. Although guest-free phases of **PCT-1** were obtained before the adsorption experiments by treatment under reduced pressure at 120 °C for 12 h and the isotherms were acquired without exposure to the atmosphere, **PCT-1** immediately adsorbed moisture from the air during XRPD measurements in ambient air.

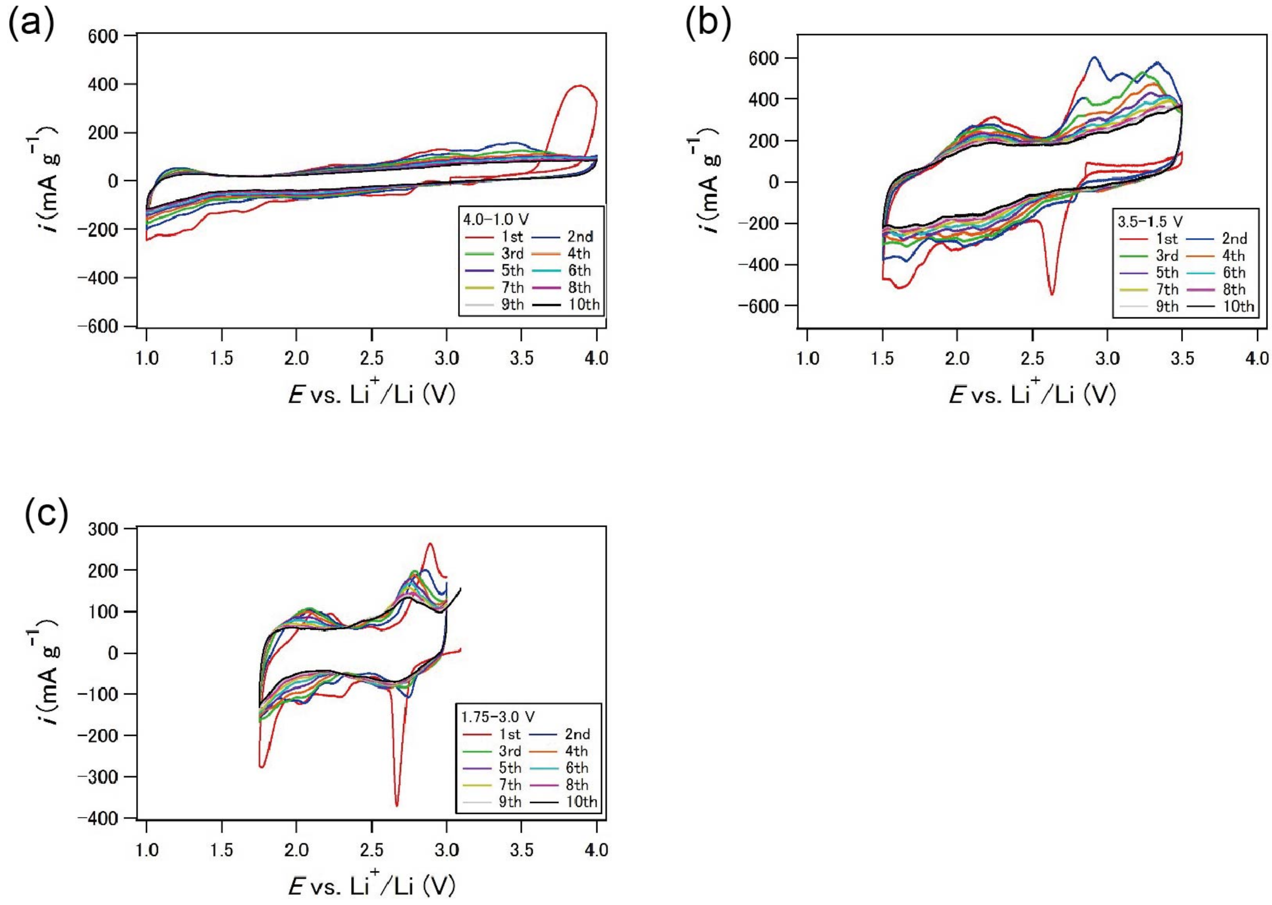

Figure S10. CV profiles for **PCT-1** cycled in various voltage ranges: (a) 1.0–4.0 V, (b) 1.5–3.0 V, and (c) 1.75–3.0 V.

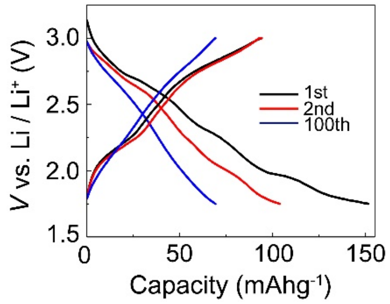

Figure S11. Galvanostatic charge–discharge profiles at 500 mA g^{-1} for **PCT-1·DMF**.

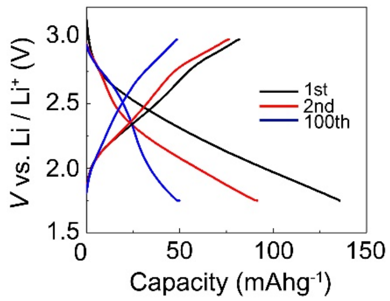

Figure S12. Galvanostatic charge–discharge profiles at 500 mA g^{-1} for **PCT-1** where the weight ratio of **PCT-1**, carbon black, and PVDF was 50:40:10.

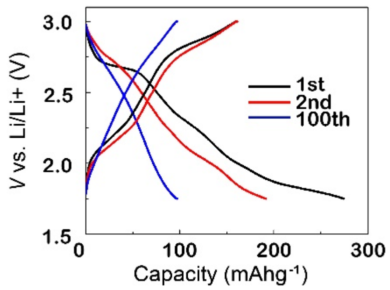

Figure S13. Galvanostatic charge–discharge profiles at 500 mA g^{-1} for **PCT-1** with a cathode of twice the thickness.

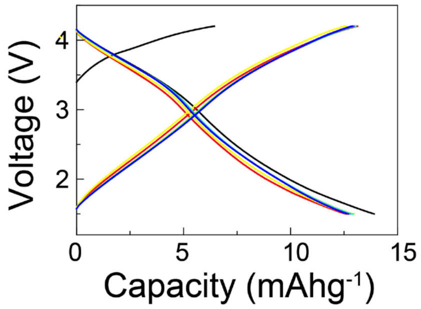

Figure S14. Galvanostatic charge–discharge profiles at 50 mA g^{-1} for a cathode without active material (only the carbon additive).

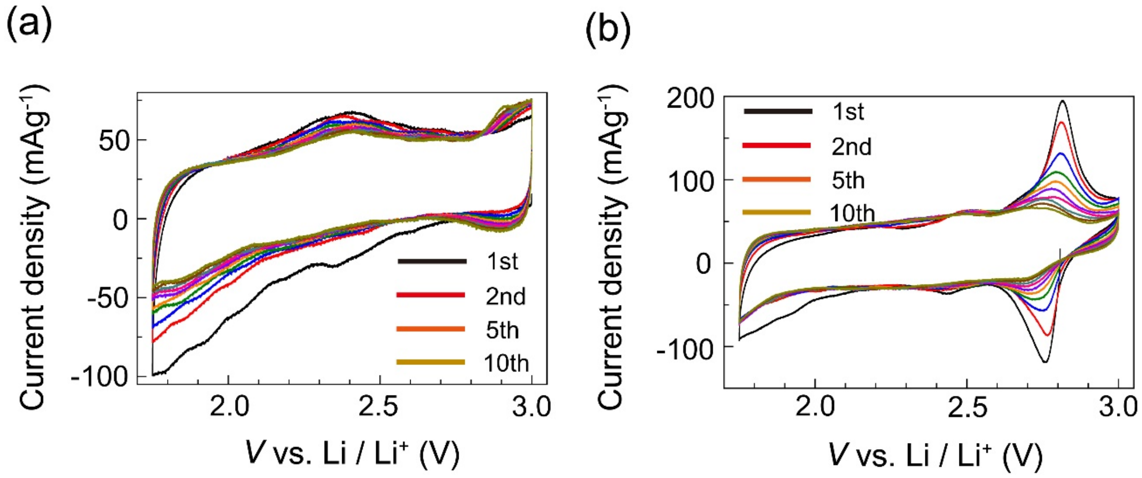

Figure S15. CV profiles for (a) HHTP and (b) HAT-CN.

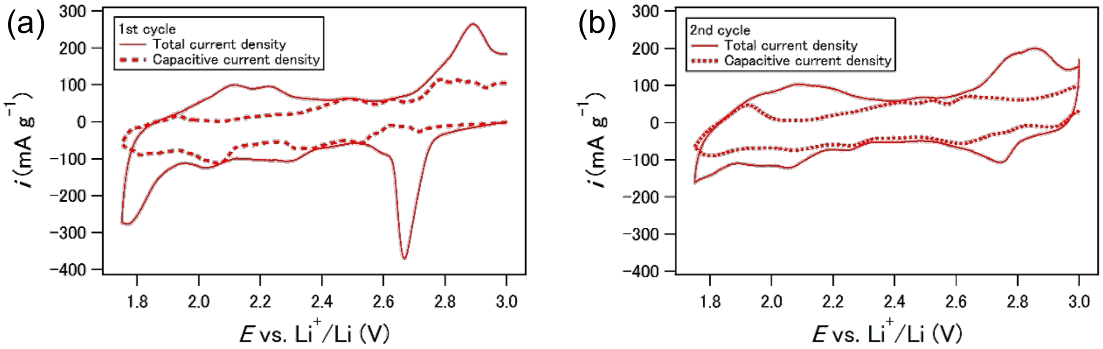

Figure S16. Total and capacitive currents during the (a) 1st and (b) 2nd CV cycles for **PCT-1**.

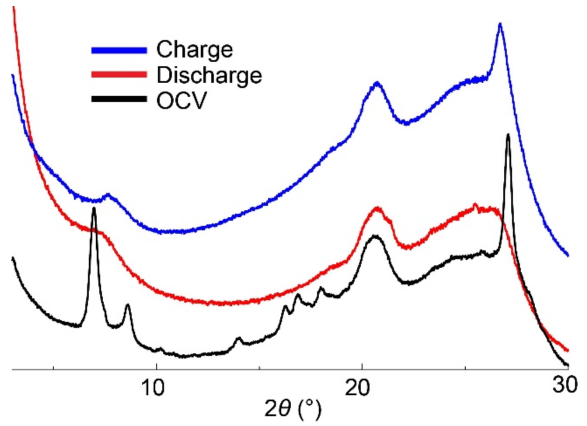

Figure S17. *Ex situ* XRPD patterns of the electrode with **PCT-1** as an active material: open-circuit voltage (OCV, black), after discharge (red), and after charge (blue).

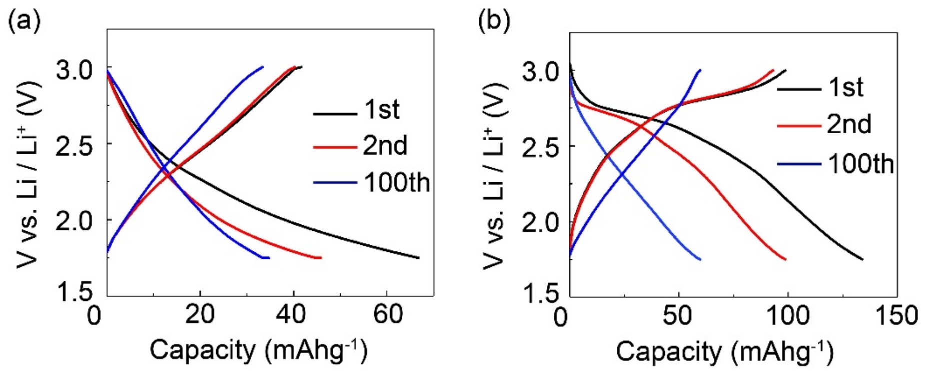

Figure S18. Galvanostatic charge–discharge profiles at 500 mA g^{-1} for (a) HHTP and (b) HAT-CN.

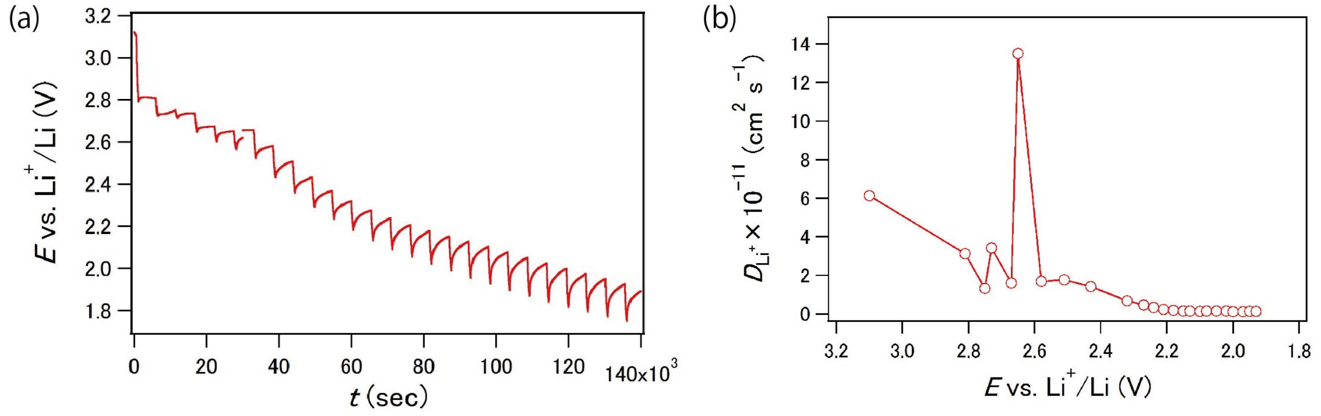

Figure S19. (a) Galvanostatic intermittent titration curve for **PCT-1** and (b) GITT diffusion coefficients at different voltages.

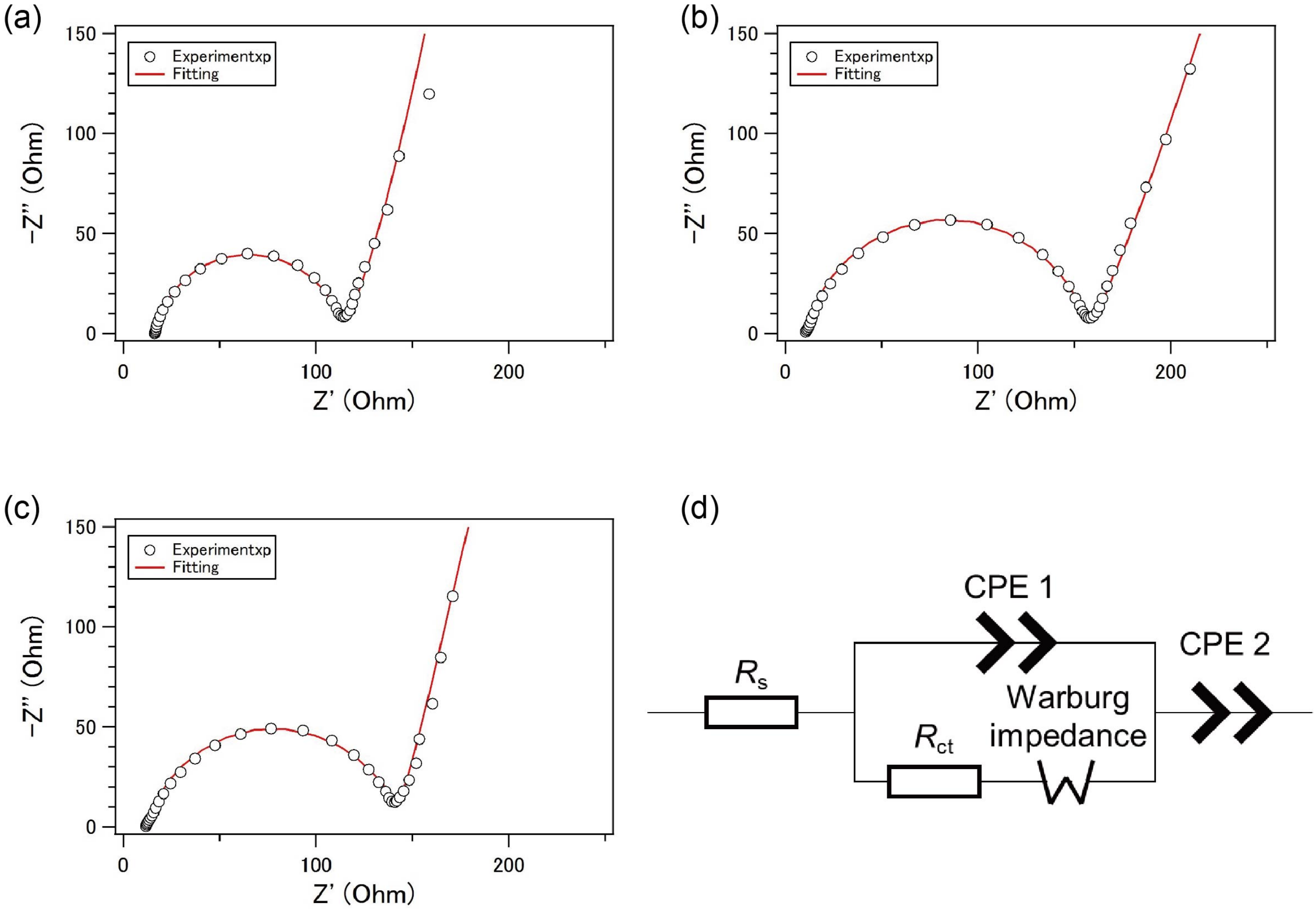

(e)

Parameters		(a) PCT-1	(b) HAT-CN	(c) HHTP
Solution resistance	R_s [Ω]	16.5	10.2	11.7
CPE 1	Q_1 [F s^{a-1}]	7.64×10^{-6}	10.5×10^{-6}	3.00×10^{-5}
	α_1	0.877	0.840	0.814
Charge transfer resistance	R_{ct}[Ω]	95.8	1.46×10^{2}	1.31×10^{2}
Warburg impedance	σ [Ω s$^{-1/2}$]	34.1	13.5	1.80
CPE 2	Q_2 [F s^{a-1}]	7.45×10^{-3}	2.58×10^{-2}	7.50×10^{-2}
	α_2	0.933	0.840	0.854

CPE

$$Z(f) = \frac{1}{Q_1 (j 2\pi f)^{\alpha_1}}$$

Warburg impedance

$$Z(f) = \frac{\sqrt{2}\sigma}{\sqrt{j 2\pi f}}$$

f : Frequency [s^{-1}]
j : imaginary unit

Figure S20. EIS profiles (circles) and the fitting results (red line) for (a) **PCT-1**, (b) HAT-CN, and (c) HHTP. (d) Equivalent circuit for fitting of the complex impedance plots. (e) Summary of the fitting parameters.

References

1. S. Grimme, *J. Comput. Chem.*, 2006, **27**, 1787–1799.
2. M. J. Frisch, G. W. Trucks, H. B. Schlegel, G. E. Scuseria, M. A. Robb, J. R. Cheeseman, G. Scalmani, V. Barone, G. A. Petersson, H. Nakatsuji, X. Li, M. Caricato, A. V. Marenich, J. Bloino, B. G. Janesko, R. Gomperts, B. Mennucci, H. P. Hratchian, J. V. Ortiz, A. F. Izmaylov, J. L. Sonnenberg, D. Williams-Young, F. Ding, F. Lipparini, F. Egidi, J. Goings, B. Peng, A. Petrone, T.

Henderson, D. Ranasinghe, V. G. Zakrzewski, J. Gao, N. Rega, G. Zheng, W. Liang, M. Hada, M. Ehara, K. Toyota, R. Fukuda, J. Hasegawa, M. Ishida, T. Nakajima, Y. Honda, O. Kitao, H. Nakai, T. Vreven, K. Throssell, J. A. Montgomery, Jr., J. E. Peralta, F. Ogliaro, M. J. Bearpark, J. J. Heyd, E. N. Brothers, K. N. Kudin, V. N. Staroverov, T. A. Keith, R. Kobayashi, J. Normand, K. Raghavachari, A. P. Rendell, J. C. Burant, S. S. Iyengar, J. Tomasi, M. Cossi, J. M. Millam, M. Klene, C. Adamo, R. Cammi, J. W. Ochterski, R. L. Martin, K. Morokuma, O. Farkas, J. B. Foresman and D. J. Fox, Gaussian 16, Revision B.01, Gaussian, Inc., Wallingford CT, 2016.

3. W. Weppner and R. A. Huggins, *J. Electrochem. Soc.*, 1977, **124**, 1569–1578.
4. Y. J. Kim, H. Kim, B. Kim, D. Ahn, J.-G. Lee, T.-J. Kim, D. Son, J. Cho, Y.-W. Kim and B. Park, *Chem. Mater.*, 2003, **15**, 1505–1511.
5. A. P. Nowak, B. Wicikowska, K. Trzciński and A. Lisowska-Oleksiak, *Procedia Eng.*, 2014, **98**, 8–13.
6. M. D. Levi, K. Gamolsky, D. Aurbach, U. Heider and R. Oesten, *J. Electroanal. Chem.*, 1999, **477**, 32–40.
7. J. Wang, J. Polleux, J. Lim and B. Dunn, *J. Phys. Chem. C*, 2007, **111**, 14925–14931.
8. E. Voisin and V. E. Williams, *Macromolecules*, 2008, **41**, 2994–2997.
9. J. T. Rademacher, K. Kanakarajan and A. W. Czarnik, *Synthesis*, 1994, **1994**, 378–380.
10. M. Kuritani, S. Tashiro and M. Shionoya, *Inorg. Chem.*, 2012, **51**, 1508–1515.
11. B. J. Smith and W. R. Dichtel, *J. Am. Chem. Soc.*, 2014, **136**, 8783–8789.
